# National rates of pediatric extremity fractures over a 20-year timespan in Denmark: a population-based descriptive cohort study

**DOI:** 10.2340/17453674.2026.45511

**Published:** 2026-03-25

**Authors:** Anja Rønnov LUND, Christian FÆRGEMANN, Per GUNDTOFT, Bjarke VIBERG

**Affiliations:** 1 Department of Orthopaedic Surgery and Traumatology, Odense University Hospital, Odense; 2 Department of Orthopaedic Surgery and Traumatology, Hospital Lillebaelt Kolding; 3 Department of Orthopaedic, Aarhus University Hospital, Aarhus, Denmark

## Abstract

**Background and purpose:**

Previous reports on incidences rates of Scandinavian pediatric extremity fractures have varied, as they are often anatomically specific and based on institution-specific findings. To gain knowledge of current and future burden on the healthcare system, a national cohort assessment is necessary. We aimed to assess the proportion and incidence within anatomical distributions of pediatric extremity fractures in relation to age, sex, and time trends.

**Methods:**

We retrieved a 20-year population-based cohort from the Danish National Patient Registry 1999–2018. We included all children aged 0–15 years with an extremity fracture diagnosis (ICD-10). We estimated fracture proportions and incidence rates (IRs) in different anatomical regions stratified by sex, age groups, and periods. IRs were estimated based on national population counts.

**Results:**

We included 668,595 pediatric fractures corresponding to an overall IR of 3,164 (95% confidence interval 3,157–3,172) per 100,000 persons/year. The highest proportion and IR were in the lower arm, but the proportions differed within the age groups. The IR increased with age and was higher in boys. The overall IR increased during the study period. In upper and lower leg fractures a decrease was seen, with all other anatomical sites increasing.

**Conclusion:**

We found an increased IR during the study period for all fractures except for the upper and lower leg. The study gives important knowledge to the healthcare system when coordinating the right resources.

The overall risk of sustaining a fracture during childhood is 10–25% [1-3]. To gain valid and sufficient information concerning the current and future burden of pediatric fractures on the healthcare system, large population-based studies are necessary.

Previous Nordic studies have demonstrated variations in the incidence rates (IRs) of childhood fractures between 163 and 255 per 10,000 persons/year (PY) [4-8]. The studies, however, were all based on local data. The time trend of pediatric fracture IRs increased in older Nordic studies with data from 1950 to 1983 [2-4]. Since 1993, different trends in IRs have been found and an increase was found in Sweden while a continuous decrease was found in Finland [4,5]. In a single local Danish study the IRs of pediatric fractures decreased by 12% from 1980–2018 [7]. Non-Scandinavian longitudinal studies have shown a decrease in the overall IR of pediatric fractures [9] or varying trends depending on anatomical area [10].

To our knowledge, no previous study has used a full national cohort to assess the time trends of pediatric fractures. Only a few studies have described IRs in more than 1 specific anatomical area, and most of these studies do not show the change in IRs over time. The aim of this study was to assess the proportion and incidence within anatomical distributions of pediatric extremity fractures in Denmark in relation to age, sex, and time trends.

## Methods

### Study design

This study is a population-based cohort study comprising all pediatric fractures in the limbs among Danish children aged 0–15 years diagnosed in any Danish hospital from January 1, 1999 to December 31, 2018. The study is reported according to the RECORD guidelines [11].

### Setting and data source

The study was conducted in Denmark with population of 5.8 million [12]. In Denmark, all individuals with permanent citizenship or residence permission are provided a unique personal identification number (PIN), which follows the individual for their entire life [13]. The PIN is used in medical records, administrative databases, and is linked to numerous national registers, which gives a unique source for data acquisition [13]. The Danish Healthcare Department offers free healthcare to everyone with a PIN.

The Danish National Patient Register (DNPR) is an administrative register with data from all public and private hospitals nationwide, going back to 1977 for all admitted patients and 1995 for all patients receiving outpatient treatment [14]. When diagnosed, all fractures are given a specific ICD-10 diagnosis code, which is mandatory in all hospitals in Denmark [15]. All ICD-10 diagnosis and procedure codes are registered longitudinally with time and date with 99.7% completeness [14]. The positive predictive value of a correct primary diagnosis in orthopedic surgery is 83%, but a specific rate has not been investigated in diagnostics of pediatric fractures [14].

### Participants and variables

The study population included all children aged 0–15 years with a Danish PIN treated in a Danish ED, outpatient clinic (OC), or orthopedic department (OD) for a limb fracture between January 1, 1999 and December 31, 2018. From the DNPR, we extracted data on all contacts with the ICD-10 codes for upper arm fractures (DS42x), forearm and wrist fractures (DS52x), hand fractures (DS62x), upper leg fractures (DS72x), lower leg fractures (DS82x), or foot fractures (DS92x) [15]. We included all types of fractures including birth fractures, open fractures, and pathologic fractures. In case of more than 1 contact with an ED, OC, or OD for the same fracture diagnosis within 3 months, only the first contact was included. Fractures sustained in another country were included if the children were referred for follow-up in a Danish ED, OC, or OD. In case of bilateral fractures with the same ICD code, only 1 of the fractures was included.

We defined 4 age groups according to the psychosocial and physiological steps in children’s development. The youngest age group were aged 0–3 years (infants), the second aged 4–7 years (early childhood), the third aged 8–11 (late childhood), and the oldest group aged 12–15 years (teenagers). The study period was divided into 4 time periods: 1999–2003, 2004–2008, 2009–2013, and 2014–2018.

### Statistics

Statistics were reported according to the guidelines of Acta Orthopaedica [16]. Population counts were extracted from statistics Denmark [17]. We extracted national sex- and age-specific mid-year population corresponding to the population at risk in each sex and age group. Based on the population counts we calculated IRs per 100,000 persons/year (PY) using the Clopper-Pearson method [15]. IRs were calculated as densities in a dynamic cohort allowing subjects to enter and leave the cohort by migration [18]. In Results all IR are presented as per 100,000/year. Children with fractures were not excluded from the population at risk. Data was presented as percentages and IR with 95% confidence intervals (CI). STATA 18 (StataCorp LLC, College Station, TX, USA) was used for all statistics.

### Ethics, funding, use of AI tools, and disclosures

Data approval was obtained (Region of Southern Denmark, jr.nr. 20/187). According to Danish legislation, no further approval was needed. No funding was obtained for this study. The authors of this study had full access to all data. AI tools were not used. BV received a research grant from SWEMAC outside this study; all other authors report no conflict of interests. Complete disclosure of interest forms according to ICMJE are available on the article page, doi: 10.2340/17453674.2026.45511

## Results

**Figure 1:**
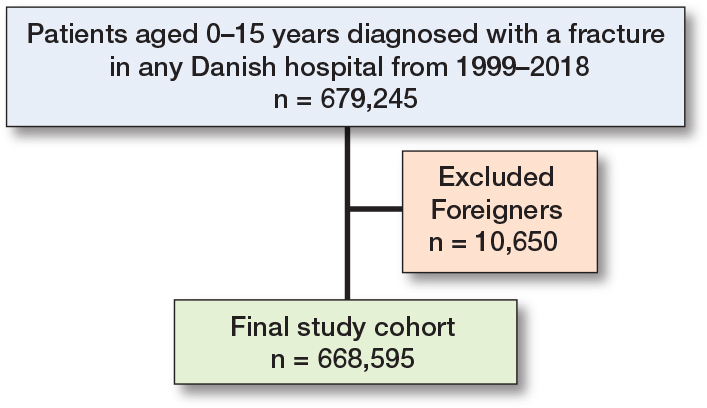
Flowchart of included patients with a pediatric fracture.

In the study period, there were 679,245 eligible patients under 16 years with a pediatric extremity fracture (Figure 1). We excluded 10,650 foreign-born individuals and included a total of 668,595 pediatric extremity fractures corresponding to approximately 33,500 fractures annually (Table 1). Boys accounted for 383,741 fractures (57%) and girls for 284,854 (43%). The median age was 10 (interquartile range [IQR] 6–13) years.

**Table 1:** Proportion in percentages of different pediatric fracture sites with 95% confidence intervals (CI) stratified by age and sex

Factor	Proportion, % (CI)						
	All, n	Upper arm	Lower arm	Hand	Upper leg	Lower leg	Foot
Sex							
Boys	383,741	14.5 (14.3–14.6)	38.0 (37.8–38.2.)	24.4 (24.2–24.4)	1.4 (1.3–1.5)	10.6 (10.5–10.7)	11.1 (11.0–11.1)
Girls	284,854	14.9 (14.8–15.0)	39.4 (39.2–39.6)	23.0 (22.8–23.2)	1.1 (1.0–1.1)	11.2 (11.1–11.3)	10.4 (10.3–10.5)
Age							
0–3	68,708	27.3 (27.0–27.7)	31.8 (31.5–32.2)	9.5 (9.3–9.7)	4.8 (4.6–4.9)	18.8 (18.5–19.1)	7.8 (7.6–8.0)
4–7	140,024	23.0 (22.8–23.2)	45.7 (45.5–46.0)	13.0 (12.8–13.2)	1.3 (1.2–1.4)	10.4 (10.3–10.5)	6.5 (6.4–6.6)
8–11	219,844	11.2 (11.0–11.4)	43.6 (43.4–43.8)	24.3 (24.1–24.5)	0.8 (0.7–0.9)	8.3 (8.2–8.4)	11.8 (11.7–11.9)
12–15	240,019	9.3 (9.2–9.4)	31.8 (31.6–32.0)	33.7 (33.5–33.9)	0.8 (0.7–0.9)	11.2 (11.0–11.4)	13.2 (13.1–13.3)
All	668,595	14.7 (14.6–14.8)	38.6 (38.5–38.7)	23.8 (23.7–23.9)	1.3 (1.2–1.4)	10.8 (10.7–10.9)	10.8 (10.7–10.9)

In total, 10% of the fractures were in the age group 0–3 years, 21% in the age group 4–7 years, 33% in the age group 8–11 years, and 36% in the age group 12–15 years (see Table 1). The highest proportions of fractures were in the lower arm (38.6%, CI 38.5–38.7) and the hand (23.8%, CI 23.7–23.9). The lowest proportion was in the upper leg (1.3%, CI 1.2–1.4). The proportions of fractures varied slightly, but significantly, with sex. The proportion of fractures varied significantly with age groups. The proportion of fractures in the upper arm and the upper leg decreased significantly with increasing age group, whereas the proportion of fractures in the hand increased significantly with increasing age group. The highest proportions of lower arm fractures were in the age groups 4–7 years and 8–11 years.

**Figure 2:**
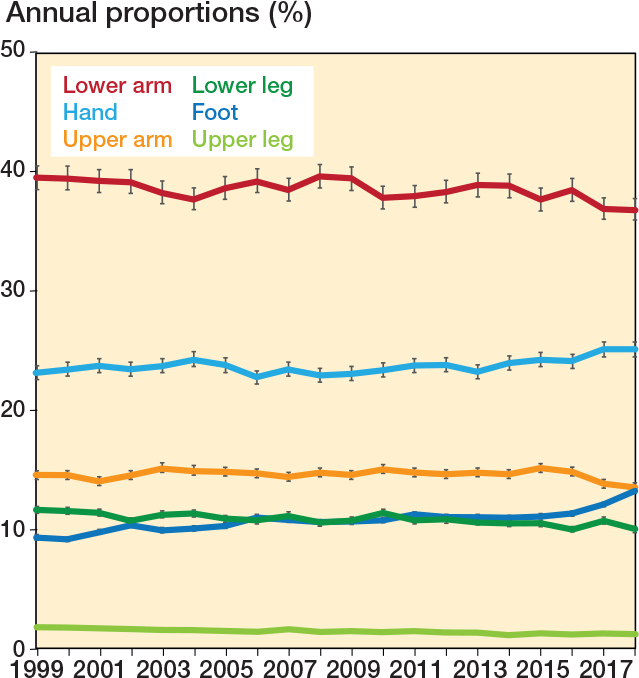
Annual proportion in percentages of different pediatric fracture sites with 95% confidence intervals.

The proportion of fractures varied slightly over time (Figure 2). The proportion of lower arm fractures, upper leg, and lower leg fractures decreased significantly in the study period, whereas the proportion of fractures in the hand and foot increased significantly in the study period.

The overall IR of extremity fractures was 3,164 (CI 3,157–3,172). For boys and girls the IRs were 3,543 (CI 3,532–3,554) and 2,766 (CI 2,756–2,776), respectively (Table 2). Lower arm fractures and fractures of the hand had the highest IRs of 1,221 (CI 1,217–1,226) and 753 (CI 749–756). Upper leg fractures had the lowest IR of 41 (CI 40–42). The IRs varied with sex and age groups (Table 2). The IRs of all fracture locations were higher in males than in females. The IRs of fractures in the hand, the foot, and the lower leg increased significantly with increasing age group whereas the IR of fractures of the upper leg decreased with increasing age group. The IR of upper arm fractures was highest in the age group 4–7 years and the IR of lower arm fractures was highest in the age group 8–11 years, at 604 (CI 598–611) and 1,778 (CI 1,766–1,789) respectively.

**Table 2:** Incidence rates of pediatric fractures sites per 100,000 persons/year with 95% confidence interval (CI) stratified by age and sex

Factor	Incidence rates 100,000 persons/year (CI)						
	All	Upper arm	Lower arm	Hand	Upper leg	Lower leg	Foot
Sex							
Boys	3,543 (3,532–3554)	512 (508–517)	1,347 (1,340–1,354)	863 (857–868)	51 (50–52)	377 (373–380)	393 (390–397)
Girls	2,766 (2,756–2776)	412 (408–416)	1,089 (1,083–1,095)	637 (632–642)	31 (30–32)	309 (306–313)	288 (284–291)
Age							
0–3	1,343 (1,333–1,353)	367 (362–373)	427 (422–433)	127 (124–130)	64 (62–66)	253 (248–257)	104 (102–107
4–7	2,625 (2,612–2,639)	604 (598–611)	1,201 (1,191–1,210)	342 (337–347)	33 (31–34)	274 (270–278)	171 (168–175)
8–11	4,078 (4,062–4,095)	456 (450–462)	1,778 (1,766–1,789)	991 (982–999)	31 (29–32)	340 (335–345)	483 (477–489)
12–15	4,538 (4,520–4,555)	422 (417–428)	1,444 (1,434–1,454)	1,529 (1,519–1,540)	38 (36–39)	506 (500–512)	599 (593–606)
All	3,164 (3,157–3,172)	463 (461–466)	1,221 (1,217–1,226)	753 (749–756)	41 (40–42)	344 (341–346)	342 (339–344)

**Figure 3:**
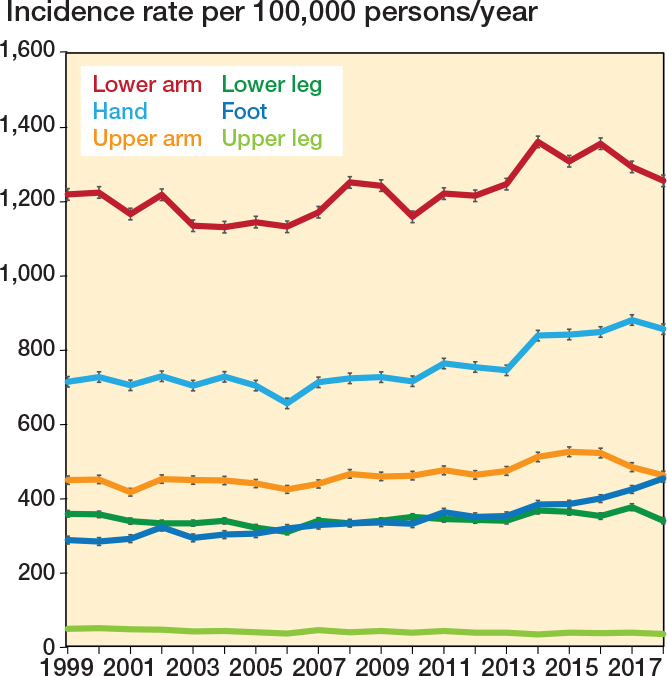
Annual incidence rates of pediatric fractures sites per 100,000 persons/year with 95% confidence intervals.

In the study period, the overall IR of limb fractures increased by 435 (CI 299-471), corresponding to a 14% increase. The IRs of upper arm, lower arm, hand, and foot fractures increased by 12 (CI 9-15), 44 (CI 40–48), 141 (CI 137–145), and 166 (CI 158–174) per 100,000 PY in the study period. The IRs of upper leg and lower leg fractures decreased by 15 (CI 12–17) and 18 (CI 14–22) in the study period (Figure 3).

## Discussion

This is the first national Danish longitudinal study of pediatric extremity fractures.

We aimed to assess proportion and incidence within anatomical distributions of pediatric extremity fractures in Denmark in relation to age, sex, and time trends. We included 668,595 pediatric fractures, corresponding to an overall IR of 3,164 (CI 3,157–3,172). The highest proportion and IR was in the lower arm and the proportions differed with age groups. The IR increased with age and was higher in boys. The IRs of upper arm, lower arm, hand, and foot fractures increased in the study period, whereas the IR of upper and lower leg fractures decreased.

The IRs were higher in our study compared with other Scandinavian studies, including a local Danish study and studies from Southern Sweden [4–8]. A study conducted with both Scandinavian data and data from Wales showed higher IRs in Wales compared with Scandinavia [19], suggesting that our finding may be more compatible to the findings from Wales rather than those from Sweden. However, another British study found lower IRs [20], thus suggesting that geography and climate may play a role in explaining the higher IRs. Another factor that could contribute to the higher IRs in other regions of the country and our study is the use of national data. Previous studies based on local data from a well-defined geographic area do not include children seeking fracture treatment at neighboring hospital outside the defined area. In our study, we included fractures from all Danish hospitals, including fractures that were sustained in other countries and followed up in a Danish hospital.

We found that the overall IR increased by 14% in the study period. A similar increase of 13% was found in a Swedish study in the period 1998–2007 [5]. Our findings do not correlate with the overall findings of a recent Danish study that found a decrease in the overall IR of pediatric fractures from a single hospital of 12% comparing the periods 1980–1984 with 2015–2018 [7]. The study also showed that the IR significantly increased from 2004–2009, which correlates with our findings of an increasing IR starting in the years 2004–2008 [7]. The age group with the highest IR was 12–15 years, which correlates with previous findings showing increasing IRs of pediatric fractures with age [4,5,7].

Our finding may be explained by a general decline in physical activity level for children over the last 25 years, as children are spending more time in front of a screen and more children are being driven to school [21,22]. The parents’ age is rising, and parents in Denmark are in general more involved in their children’s lives and activities. The average distance for an individual to an ED in Denmark is estimated to be 20 km and there is no payment to be assessed. We believe that the increase of fractures over time in the present study could be due to the children now being more likely to get to an ED for an examination rather than being a net increase in actual fractures.

Even though the highest overall proportions and IRs of fractures were in the lower arm, the peak age group for lower arm fractures was 8–11 years, whereas fractures in the hand were more common in the 12–15 years age group compared with lower arm fractures. Previous studies have all shown that lower arm fractures are the most common fractures in children, but none of these studies show the proportions within the age groups [4,5,7,8]. None of the anatomic sites alone were responsible for the overall increase in IR in our study in the study period, but the most pronounced increase in fractures was found in the hand and foot, which increased by 19% and 38%, respectively in the study period.

### Strengths

One strength of this study is the long study period with continuous data. Second, we had access to national data from all hospitals in Denmark and access to valid mid-year population counts from Statistics Denmark. All ICD-10 diagnosis codes were registered longitudinally with time and date with 99.7% completeness [14].

### Limitations

Bias may arise due to coding misclassifications or errors including changes in the coding procedures in the study period. We have no valid information concerning changes in the registration in each hospital in the 20-year period. Fracture diagnosis coding in children has not been validated. In the study period, the Danish healthcare system has changed as some EDs have changed from open access to referred access ED. However, the alteration mainly affected patients with minor injuries and not children with bone fractures [23]. The IRs described include only cases requiring medical attention in Danish hospitals. We have no information available regarding the number of cases who seek medical attention at the general practitioners or in hospitals outside Denmark. However, in Denmark, general practitioners do not treat fractures. Furthermore, most Danish children sustaining fractures in other countries are referred to follow-up visits in the Danish hospitals and are therefore included in our study. We did not include birth fractures coded with ICD-codes DP13x, which include birth lesions in the bones. This bias may lead to an underestimation of the frequency and IR of fractures in the youngest age group but we believe it is minor. The DNPR contains no reliable information regarding the side of the fracture (right, left, bilateral). In the case of bilateral fractures with the same ICD code, only 1 of the fractures is registered in the DNPR. This may lead to underestimation of the frequency and IR of some fractures. We believe this has limited impact on the results. In the study, we included a 3-month wash-out period as we included only the first contact with the same diagnosis within 3 months. This may lead to underestimation of repeat fractures, especially as a previous study has shown that refractures can occur 2 years after the primary fracture; however, the overall refracture rate in this study was only 0.48%, making refractures less significant in our study [24]. We have no reliable information in the DNPR on whether recurrent contacts with the ED, OC, or OD are due to follow-up contacts or new fracture contacts.

### Conclusion

The highest proportion and IR were in the lower arm, but the proportions differed between the age groups. The IR increased with age and was higher in boys. The overall IR increased during the study period.

We provide a baseline for comparing Danish data with neighboring countries and for evaluation of future interventions. Furthermore, the study gives important knowledge to the healthcare system when coordinating the right resources.

### Supplementary data

Supplementary Tables 1–2 with annual incidence rates and CI according to detailed anatomical sites stratified by age group are available on the article page, doi: 10.2340/17453674.2026.45511

## Supplementary Material



## Notes

ARL: formal analysis, investigation, writing—original draft, writing—review and editing. CF: formal analysis, methodology, supervision, writing—original draft, writing—review and editing. PG: formal analysis, methodology, project administration, resources, writing—original draft, writing—review and editing. BV: conceptualization, formal analysis, methodology, project administration, resources, supervision, writing—original draft, writing—review and editing.

Handling co-editors: Ilkka Helenius and Robin Christensen

*Acta* thanks Torsten Backteman, David Farrington and Johan von Heideken for help with peer review of this manuscript.
