# Supplementary material for: National rates of pediatric extremity fractures over a 20-year timespan in Denmark: a population-based descriptive cohort study
Source: Acta Orthop. 2026 Mar 25;97:204–8. doi: 10.2340/17453674.2026.45511 (PMC13019125; doi:10.2340/17453674.2026.45511)
Supplement: Supplementary file 1 [file ActaO-97-45511-s1.pdf]

Supplementary Table 1. Annual incidence rates including 95% confidence intervals (CI) of upper limb fractures according to anatomical site stratified by age group

Fracture site	Diagnosis	Incidence per 100,000 persons/year (CI)			
	ICD-10 codes (DK)	0–3 years	4–7 years	8–11 years	12–15 years
Upper arm					
Clavicle	S420	194 (190–198)	200 (197–204)	127 (124–130)	208 (204–212)
Proximal humerus	S422	26 (24–28)	55 (53–57)	92 (89–96)	75 (73–77)
Shaft of humerus	S423	17 16–18)	20 (19–21)	22 (20–22)	17 (15–18)
Distal humerus	S424	123 (120–126)	316 (311–321)	203 (199–207)	100 (98–102)
Other/unspecified [a]	S421, S427, S428, S429	7.3 (6.6–8.0)	13 (12–14)	13 (12–14)	23 (22–24)
Forearm					
Proximal radius/ulna	S520, S521	48 (46–50)	130 (127–133)	160 (156–163)	136 (133–139)
Shaft of radius/ulna	S522, S523, S524	106 (103–108)	251 (247–255)	263 (259–267)	196 (192–200)
Distal radius/ulna	S525, S526, S528	245 (241–250)	742 (735–750)	1259 (1250–1269)	1030 (1022–1039)
Other/unspecified	S527, S529	28 (26–30)	76 (74–78)	96 (93–98)	81 (79–84)
Hand					
Scaphoid bone	S620	1.2 (0.9–1.5)	5.3 (4.7–5.9)	151 (148–154)	299 (295–304)
Other carpal bone	S621	0.8 (0.6–1.1)	2.9 (2.5–3.4)	17 (16–18)	29 (28–30)
Metacarpal bone	S622, S623, S624	14 (13–15)	48 (46–50)	170 (166–173)	342 (337–347)
Finger	S625, S626, S627	105 (102–107)	266 (262–271)	607 (601–614)	806 (798–813)
Other/unspecified	S628	7.1 (6.4–7.8)	20 (19–21)	46 (44–48)	53 (51–55)

[a] Including fractures of scapula.

Supplementary Table 2. Annual incidence rate including 95% confidence intervals (CI) of lower limb fractures according to anatomical site stratified by age group

Fracture site	Diagnosis	Incidence per 100,000 persons/year (CI)			
	ICD–10 codes (DK)	0–3 years	4–7 years	8–11 years	12–15 years
Upper leg					
Proximal femur	S720, S721, S722	9.3 (8.5–10.2)	4.7 (4.1–5.3)	6.5 (5.9–7.2)	11 (10–12)
Shaft of femur	S723	36 (34–38)	18 (17–19)	13 (12–14)	11 (10–12)
Distal femur	S724	11 (10–12)	7.1 (6.5–7.9)	7.1 (6.4–7.8)	10 (9–11)
Other/unspecified	S727, S728, S729	7.5 (6.8–8.3)	3.4 (2.9–3.9)	3.6 (3.1–4.2)	5.0 (4.8–5.7)
Lower leg					
Proximal tibia/patella	S820, S821	42 (40–44)	27 (26–28)	31 (30–33)	58 (56–60)
Shaft of tibia/fibula	S822, S824	75 (73–78)	74 (72–76)	41 (39–43)	41 (39–43)
Distal tibia	S823	64 (62–66)	39 (37–41)	38 (37–39)	70 (68–72)
Ankle	S825, S826, S827x, S828x	25 (24–26)	92 (90–95)	184 (181–188)	259 (254–263)
Other/unspecified	S827, S828, S829	47 (45–49)	42 (40–44)	45 (44–47)	78 (76–80)
Foot					
Calcaneus	S920	2.2 (1.8–2.6)	4.7 (4.2–5.4)	12 (11–13)	9.5 (8.7–10.3)
Talus	S921	0.8 (0.6–1.0)	2.5 (2.1–3.0)	5.5 (4.9–6.1)	9.5 (8.7–10.3)
Other tarsal bone	S922	3.5 (3.0–4.0)	67 (6.1–7.4)	20 (19–21)	24 (23–26)
Metatarsal bone	S923	70 (67–72)	85 (82–87)	178 (174–181)	207 (203–211)
Toes	S924, S925	25 (23–26)	67 (65–69)	251 (247–256)	328 (323–333)
Other/unspecified	S926, S927, S929	3.9 (3.3–4.2)	5.8 (5.2–6.4)	17 (16–18)	22 (21–23)
